# The Ouranos CRCM5-CMIP6 ensemble: A dynamically downscaled ensemble of CMIP6 simulations over North America

**DOI:** 10.1038/s41597-025-06289-7

**Published:** 2025-12-12

**Authors:** Dominique Paquin, Christopher D. McCray, Charles B. Gauthier, Michel Giguère, Olivier Asselin, Pascal Bourgault, Marie-Pier Labonté, Dominic Matte

**Affiliations:** Ouranos, Montréal, Québec Canada

**Keywords:** Climate change, Climate and Earth system modelling, Atmospheric science

## Abstract

This paper presents Ouranos’ contribution to the North American component of the Coordinated Regional Climate Downscaling Experiment (NA-CORDEX). The fifth-generation Canadian Regional Climate Model (CRCM5) is driven by four global models from the sixth phase of the Coupled Model Intercomparison Project (CMIP6). Simulations are produced at 0.11° horizontal grid spacing over North America from 1950 to 2100. Up to four different emissions scenarios are used for future climate simulations (2015–2100). Five ensemble members are available for a selected model and scenario. Here, we describe the model, the chosen configuration, model validation and provide instructions for accessing the data.

## Background & Summary

The World Climate Research Program (WCRP) Coordinated Regional Climate Downscaling Experiment (CORDEX) project aims to advance and coordinate the science and application of regional climate downscaling through global partnerships. Among the goals of CORDEX, is the production of coordinated sets of regional downscaled projections worldwide. It is not expected that each modeling group will produce simulations driven by each GCM, but rather that a common protocol will be followed, allowing users to combine multiple ensembles. North America (NA) is one of 14 different domains on which different groups are solicited to produce regional climate information through numerical or empirical statistical downscaling. This paper presents Ouranos’ dynamical downscaling contribution to the second phase of NA-CORDEX using the fifth-generation Canadian Regional Climate Model (CRCM5)^1,2^ driven by selected CMIP6 models.

We briefly describe the model and present the chosen configuration for this operational ensemble, including selected outputs. The motivations behind the CMIP6 model selection are discussed. Access to data, via THREDDS and Globus is presented. We conclude the paper with our standard validation, which is done to detect potential problems with individual simulations, to quantify structural biases, to characterize the signal coming from the driving models and emission scenarios, and to evaluate the climate change signal.

## Methods

### Model description

The CRCM5 is a regional climate model (RCM) developed by the Centre pour l’étude et la simulation du climat à l’échelle régionale (ESCER) at the Université du Québec à Montréal (UQAM), in partnership with Environment and Climate Change Canada (ECCC) and Ouranos. This RCM is based on the GEM (Global Environmental Multiscale) model version 3.3.3.1, a numerical weather prediction model^3^. The model uses a rotated limited-area grid and a semi-implicit semi-Lagrangian numerical scheme. The model is coupled to the Canadian Land Surface Scheme (CLASS) version 3.5 and to the Freshwater Lake (FLake) model for subgrid lakes^4^. The following parameterizations are used: Kain-Fritsch deep moist convection^5^; Kuo-transient shallow convection^6^,^7^; Sundquist large-scale condensation^8^; Bourgouin precipitation phase partitioning^9^; Li & Barker radiation^10^. Prescribed non-evolving aerosols and prescribed ocean sea surface temperature (SST) and sea ice fraction are used. Martynov *et al*.^1^ provide a detailed description of CRCM5 along with an assessment of its biases relative to various observational products.

### Configuration

**Fig. 1:**
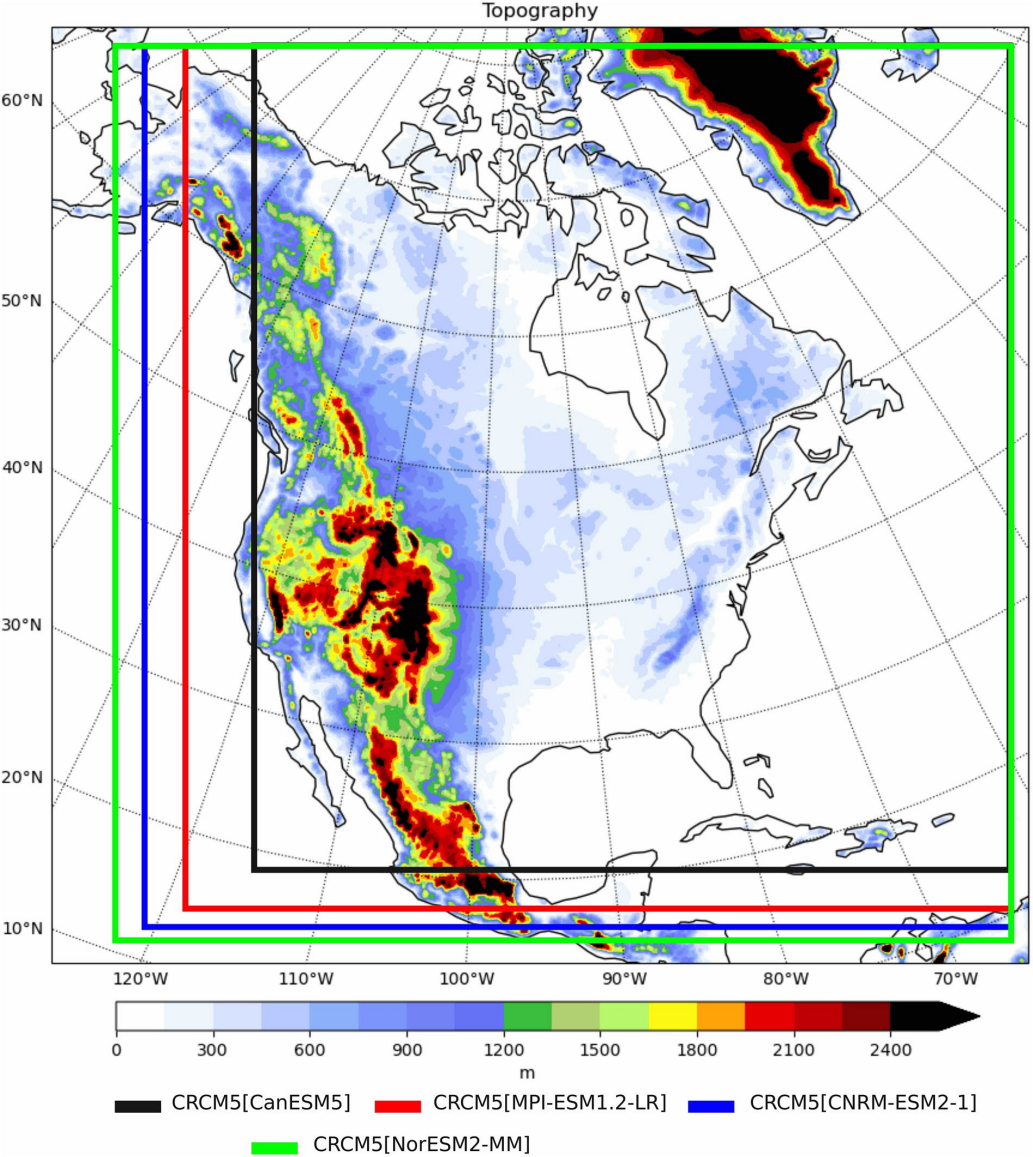
Topography for Ouranos’ CRCM5-CMIP6 free domain over the North American CORDEX domain. The coloured boxes show the recommended exclusion zone according to the resolution of the four selected CMIP6 driving models.

The simulations are computed over a domain covering North America, following the CORDEX protocol. The domain consists of 695 × 668 grid points, including a 10-point Davies sponge zone—where the driving fields are merged toward the RCM solution following a ${\cos }^{2}$ function—and a 10-point halo zone surrounding the domain. This gives a free zone for analysis of 655 × 628 grid points where the solution is fully provided by the RCM, shown in Fig. 1. The horizontal mesh size is 0.11° on a rotated latitude-longitude grid (about 12 km resolution). The timestep is 5 minutes. Fifty-six hybrid vertical layers are used in the atmosphere, including 11 below 1 km, the lowest being at 30 m above surface. Three-dimensional fields are converted to pressure levels prior to archival. Data are archived hourly or every 3 hours depending on the variable. The surface scheme performs calculations on 17 levels down to 15 m below ground. The thickness of each soil layer (from the surface) is : 0.1, 0.2, 0.3, 0.4, 0.5 (x10), 1.0, 2.0 and 3.0 m. Geophysical fields are listed on Table 1.

**Table 1:** Sources for the geophysical fields used in the CRCM5-CMIP6 ensemble.

Variable	Source	Native Resolution
Soil texture	ECOCLIMAP^28^	1 km
Bedrock depth	Shangguan^29^	≈30 m × 30 m
Land cover	USGS-GLCC^30^	900 m
Topography	GTOPO30^31^	900 m

A smooth spectral nudging of the large scales^2,11^ is applied to the horizontal wind components within the RCM domain. The spectral nudging configuration consists of large-scale features with a half-response wavelength of 1177 km and a relaxation time of 13.34 h. These large scales are imposed inside the RCM domain and vary along the vertical: the nudging strength is set to zero from the surface to a height of 500 hPa and increases linearly upward to the top of the model’s simulated atmosphere (10 hPa).

Computations are done on the Beluga or Narval supercomputers using 19×24 or 19×20 nodes. Model output is written in ECCC’s standard binary format. A four-step post-production process is applied to the model output: (1) conversion from binary to NetCDF format, (2) calculation of secondary variables such as radiative balance and soil water integration, (3) monthly averaging, and (4) monthly archiving of variables following chunking. Time series and monthly means are transferred to Ouranos’ archival system. An additional compression is done prior to transfer to the THREDDS server by using a “bit-rounding” method and retaining 12 bits^12^.

Following CORDEX experiment design, we carry out an evaluation simulation driven by the fifth-generation European Centre for Medium-Range Weather Forecasts (ECMWF) Reanalysis (ERA5)^13^ for the period 1979–2020. ERA5 is a global reanalysis product at 0.25° grid spacing. We also produced a second ERA5-driven member initialized one day later than the first to explore the internal variability of the CRCM5, which is the smallest component of the total variability among many factors including emission scenarios, driving GCMs and natural variability^14^. Results presented here use only the first member, as differences between the two members are extremely small, with a mean temperature difference for 1981–2000 of 0.005 K.

Simulations driven by GCMs from the Coupled Model Intercomparison Project 6 (CMIP6) cover years 1950–2014 for the historical period, while future climate simulations span 2015–2100. Future simulations follow several shared socioeconomic pathway (SSP) climate scenarios^15^. SSP1-2.6 and SSP3-7.0 are required by CORDEX. Simulations following SSP2-4.5 are also run given interest in this scenario for climate adaptation. One simulation following the high-emissions SSP5-8.5 scenario has also been carried out.

### Driving data

ERA5 data are downloaded from the ECMWF web site. The CMIP6 driving data (Table 2) are downloaded from the Earth System Grid Federation (ESGF) nodes using wget or via globus. All data are converted to ECCC standard format prior to computing. The driving data consist of 6-hourly specific humidity (HU), temperature (TA), wind components (UA and VA) on model levels, surface pressure (PS), daily sea-surface temperature (TOS) and sea-ice concentration (SICONC), and topography (OROG).

**Table 2:** Details of driving data used for simulations. ERA5-driven simulations are performed to evaluate structural biases and exploring internal variability by comparing the two members starting one day apart. Members driven by different global climate models allow for analysis of future climate change.

Global data	Period	Emissions	Members
ERA5^13^	1979-2020	observed	2
CanESM5^32^	1950-2014	observed	1
CanESM5	2015-2100	SSP1-2.6	1
CanESM5	2015-2100	SSP2-4.5	1
CanESM5	2015-2100	SSP3-7.0	1
CanESM5	2015-2100	SSP5-8.5	1
MPI-ESM-1-2-LR^33^	1950-2014	observed	5
MPI-ESM-1-2-LR	2015-2100	SSP1-2.6	1
MPI-ESM-1-2-LR	2015-2100	SSP2-4.5	1
MPI-ESM-1-2-LR	2015-2100	SSP3-7.0	5
CNRM-ESM2-1^21^	1950-2014	observed	1
CNRM-ESM2-1	2015-2100	SSP1-2.6	1
CNRM-ESM2-1	2015-2100	SSP2-4.5	1
CNRM-ESM2-1	2015-2100	SSP3-7.0	1
NorESM2-MM^34^	1950-2014	observed	1
NorESM2-MM	2015-2100	SSP1-2.6	1
NorESM2-MM	2015-2100	SSP2-4.5	1
NorESM2-MM	2015-2100	SSP3-7.0	1

We have selected four of the 56 models with data submitted to CMIP6 ScenarioMIP : CanESM5, CNRM-ESM2-1, MPI-ESM-1-2-LR and NorESM2-MM. Although selecting only four models out of 56 may seem like a very small sample, most are not suitable for RCM application due to technical or compatibility constraints. Moreover, many of these models are interdependent (see^16^), which further limits the effective number of independent simulations to be selected. To elaborate further, models are selected based on several criteria, including data availability, model performance, climate sensitivity and compatibility with the CRCM5. The most important criterion is the availability of the data needed to drive the simulations. We require driving data for the historical simulation, which is an entry deck for CMIP6, and future simulations from the ScenarioMIP project. Data for SSP1-2.6, SSP2-4.5, and SSP3-7.0 must be available. As CRCM5 can handle only Gregorian or fixed 365-day calendars, models with a fixed 360-day calendar are excluded. We also attempted to span a wide range of equilibrium climate sensitivity (ECS) values, which represent the amount of long-term warming resulting from a doubling of atmospheric CO_2_ concentrations. The ECS values for our selected driving GCMs include 5.64 K for CanESM5, 4.8K for CNRM-ESM2-1, 3.0K for MPI-ESM-1-2-LR and 2.54 K for NorESM2-MM. Additionally, more subjective selection crtieria include storage space required for the driving data (related to model resolution), collaborations with model developers (CanESM5), and use of the model by other regional modelling communities such as EURO-CORDEX (CNRM-ESM2-1). The possibility of using several members favored the selection of MPI-ESM-1-2-LR. Performance of the driving GCMs globally and over North America was also considered by comparing key driving variables to ERA5, including temperature, specific humidity, mean and variance of geopotential, sea surface temperature, and sea ice extent (e.g. Model Selection Dashboard. However, this has been used only for the selection of last GCM (NorESM2-MM).

As identified by Matte *et al*.^17^, the differing resolutions of the driving GCMs requires exclusion zones, also called spatial spin-up zones, within the domain where the RCM develops its own small scales allowed by the higher resolution. Those zones are situated on the side of the domain most influenced by the inflow circulation. Consequently, the excluded spin-up zone lies primarily along the western and southern boundaries, and its width depends on the resolution of the driving data-explaining why CanESM5 exhibits the largest spin-up zone^17^. Those data within these zones near the edges of the domain should be excluded. Zones for each of the driving GCMs are displayed in Fig. 1.

### Model output

A total of 84 output variables were selected among hundreds of possibilities, following CORDEX recommendations and operational needs at Ouranos. A comprehensive list of these output variables along with a short description, archival frequency, realm and type is available at 10.5281/zenodo.17465777^18^. The possible variable types are: instantaneous values (I) provided every archival time, mean value (M) among all time steps since the last archive and miNimum (N) or maXimum (X) values between archival times. For variable types M, N and X, the values of the time dimension correspond to the center of the time interval over which the minimum, maximum or average is calculated. For instance, the precipitation flux (pr) is given at times 0:30, 1:30, 2:30, etc., which correspond to the average precipitation flux over the time intervals 0:00-1:00, 1:00-2:00, 2:00-3:00, etc., respectively. The latter interval bounds are provided via the variable time_bnds present in the time series of variables of type M, N or X. Daily and monthly means are calculated from hourly and 3-hourly output. For some variables, only monthly means are archived. For 3D atmospheric variables, data is provided on 11 pressure levels : 1000, 975, 950, 925, 900, 850, 800, 700, 600, 500 and 250 hPa. For 3D soil variables, data is provided at the 17 soil levels down to a depth of 15 m. However, variables related to soil moisture should be used with caution below a depth of 2 m, because deep-soil moisture cannot be considered to be in equilibrium in this ensemble.

## Data Records

The reference associated with the CRCM5-CMIP6 simulation ensemble is 10.5281/zenodo.17465777^18^. Out of a total of 84 output variables, 45 variables are (status T) available through a public Thematic Real-time Environmental Distributed Data Services server (THREDDS). The selection of variables shared publicly is based on the CORDEX-CMIP6 data request. The THREDDS dataset are accessible at the following links:

- for monthly:https://pavics.ouranos.ca/twitcher/ows/proxy/thredds/catalog/datasets/simulations/RCM-CMIP6/CORDEX/NAM-12/mon/catalog.html
- for daily: https://pavics.ouranos.ca/twitcher/ows/proxy/thredds/catalog/datasets/simulations/RCM-CMIP6/CORDEX/NAM-12/day/catalog.html
- for 3-hourly:https://pavics.ouranos.ca/twitcher/ows/proxy/thredds/catalog/datasets/simulations/RCM-CMIP6/CORDEX/NAM-12/3hr/catalog.html
- for hourly: https://pavics.ouranos.ca/twitcher/ows/proxy/thredds/catalog/datasets/simulations/RCM-CMIP6/CORDEX/NAM-12/1hr/catalog.html

The simulations hosted on THREDDS are stored in NetCDF format. Each aforementioned link contains 14 NetCDF files, corresponding to one global model, period and emission scenario combination (Table 2). All variables are simulated over the North American domain at 0.11° (approximately 12 km) grid spacing on a rotated latitude-longitude grid. The attributes of the crs variable in each NetCDF provide details of the grid.

## Technical Validation

### Structural bias

**Fig. 2:**
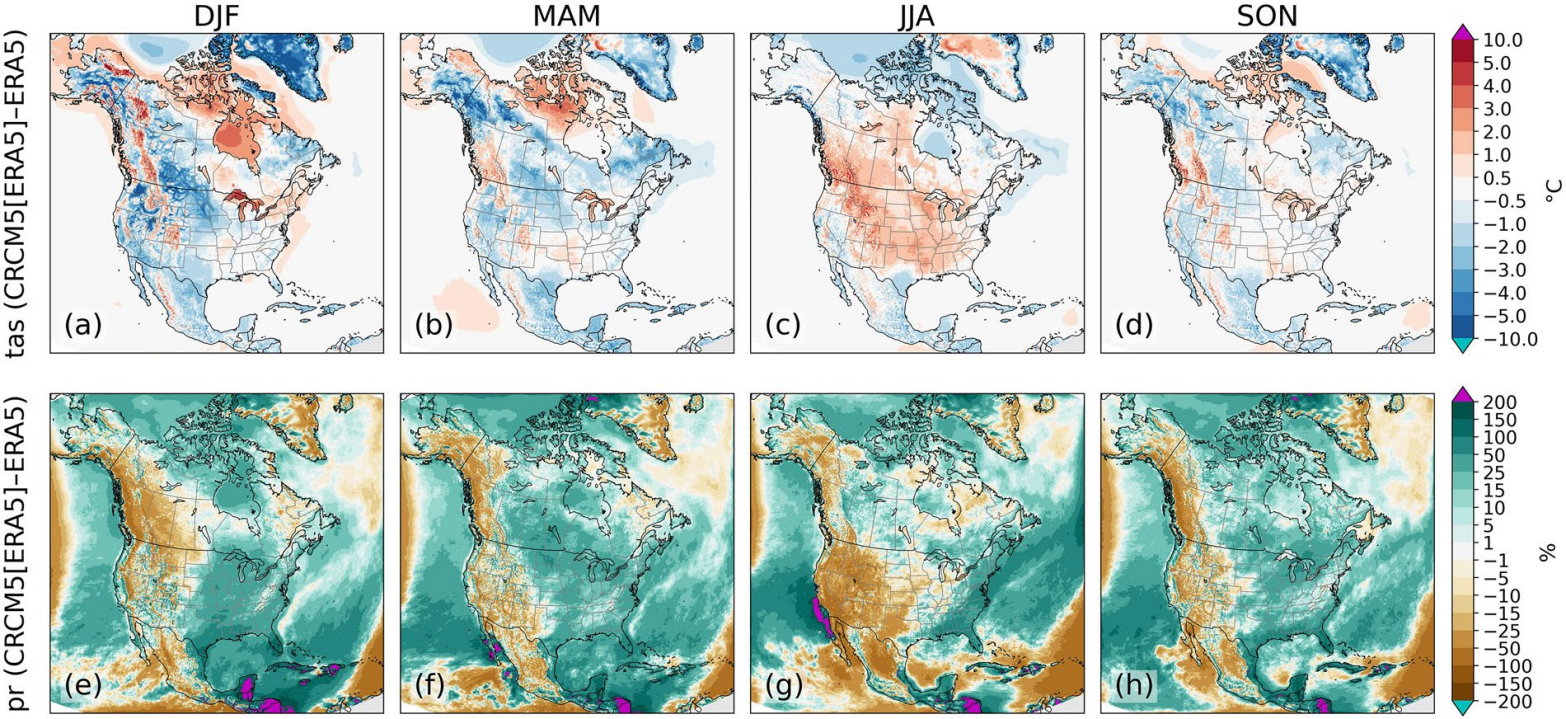
CRCM5 structural biases for temperature (tas, **a**–**d**) (°C) and precipitation (pr, **e**–**h**) (relative changes in %) for winter (**a,e**), spring (**b,f**), summer (**c,g**) and autumn (**d,h**) months. Biases are calculated by subtracting 1981–2000 seasonal means from ERA5 from means from the CRCM5 simulation driven by ERA5 (member 1) over the same period.

To explore the structural biases in the CRCM5, we first compare results from the simulation driven by ERA5 with output from ERA5 itself. Temperature biases are relatively small except for a strong wintertime cold bias over Greenland and a weaker bias over portions of the southern Canadian and northern U.S. Plains, from Alberta and Manitoba southward to Minnesota and South Dakota (Fig. 2a). A summer warm bias (≈0.5–3 °C) is present over much of the United States and western Canada (Fig. 2c). In spring, a cold bias is present over a broad region corresponding to the boreal forest regions from Alaska southeastward to Quebec and Labrador (Fig. 2b). Biases in the autumn are generally less pronounced (Fig. 2d).

An east-west gradient is present in the precipitation bias, with wet biases over the eastern portion of the continent and dry biases from the Rocky Mountains westward (Fig. 2e–g). Wet biases on the order of 10-50%, corresponding to absolute biases of 0.5–2 mm day^−1^, are present over the Eastern U.S. and southeastern Canada in all seasons, though their spatial extent is smaller in summer (Fig. 2g). Conversely, the dry bias is strongest in summer, extending from California eastward across the U.S. Plains. A narrow east-west band of dry biases are also present in every season at the westernmost boundary of the domain, corresponding to the approximate locations of the North Pacific storm track. These biases are associated with the exclusion zone discussed in the Methods section.

These results are very similar to those found by Martynov *et al*.^1^ who compared a lower resolution (0.44°) CRCM5 simulation driven by the previous generation ECMWF reanalysis (ERA-Interim^19^). This is unsurprising given the minimal differences between the two CRCM5 configurations, although benefits are revealed through the analysis of weather phenomena^20^.

### Evaluation of GCM-driven simulations

**Fig. 3:**
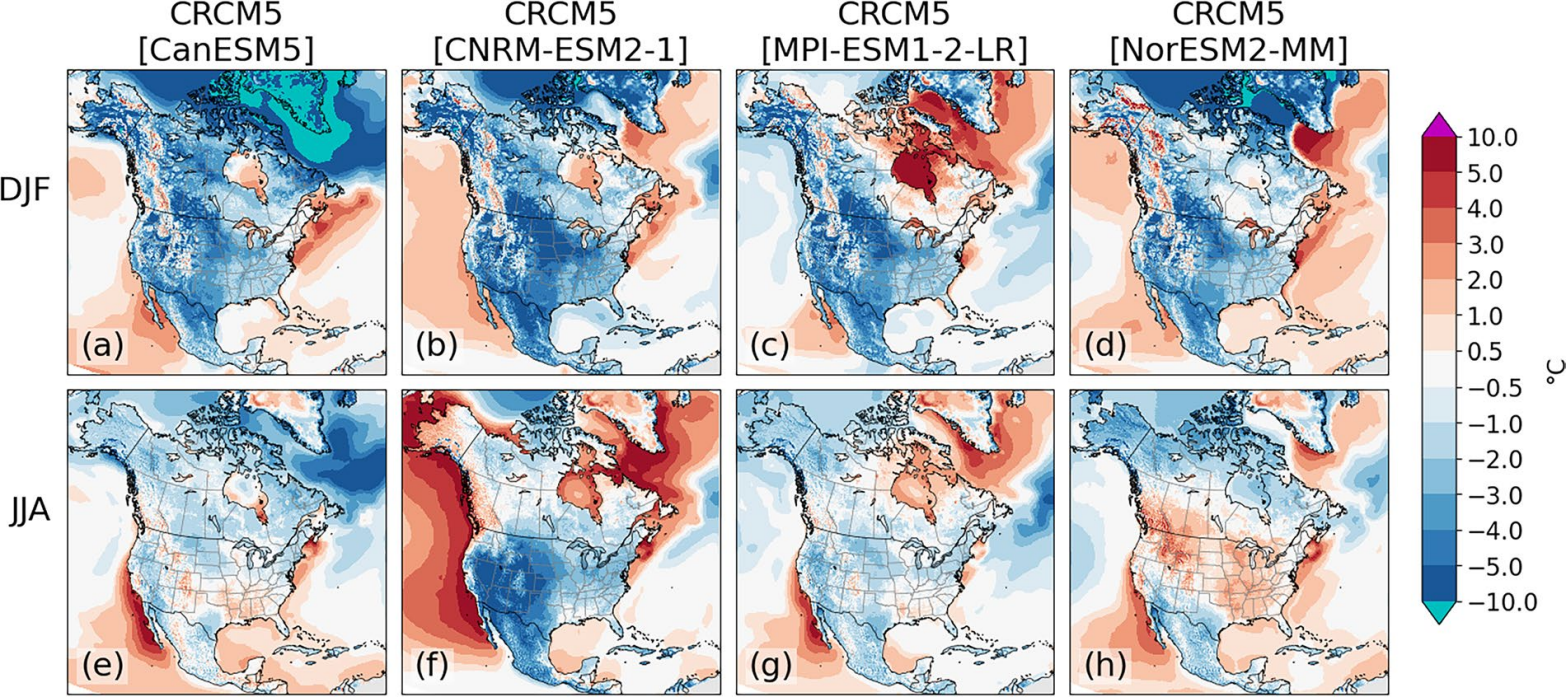
CRCM5 temperature biases (°C) for winter (**a**–**d**) and summer (**e**–**h**) for the historical climate simulations driven by CanESM5 (**a**), CNRM-ESM2-1 (**b**), MPI-ESM1-2-LR (**c**) and NorESM2-MM (**d**). Biases are calculated by subtracting 1971-2000 seasonal means from the simulation from those of ERA5.

The validation process for simulations driven by GCMs involves similar comparisons between the historical simulations and ERA5 over a common period, with 1971–2000 presented here. Spatial patterns of key variables like daily average, maximum and minimum temperature (tas, tasmax and tasmin) and precipitation (pr) are explored to ensure the model presents physically reasonable representations of the climate. Biases in this section are primarily a combination of the structural biases presented above, and the biases inherited from the driving GCMs. For temperature, most regions present a cold bias in winter, with a slight warm bias in the CRCM5[MPI-ESM1-2-LR] simulation over portions of eastern and northern Canada (Fig. 3c). The strongest cold biases, sometimes exceeding 5 °C, are found in winter over the U.S. Northern Plains and the Canadian Prairies (Figs. 3a–d). Biases in summer are weaker for all simulations except CRCM5[CNRM-ESM2-1], which has a strong cold bias over the Western U.S. and strong warm biases over the East Pacific, Labrador Sea and North Atlantic near Greenland (Fig. 3f). These biases are consistent with those of the driving GCM itself^21^.

**Fig. 4:**
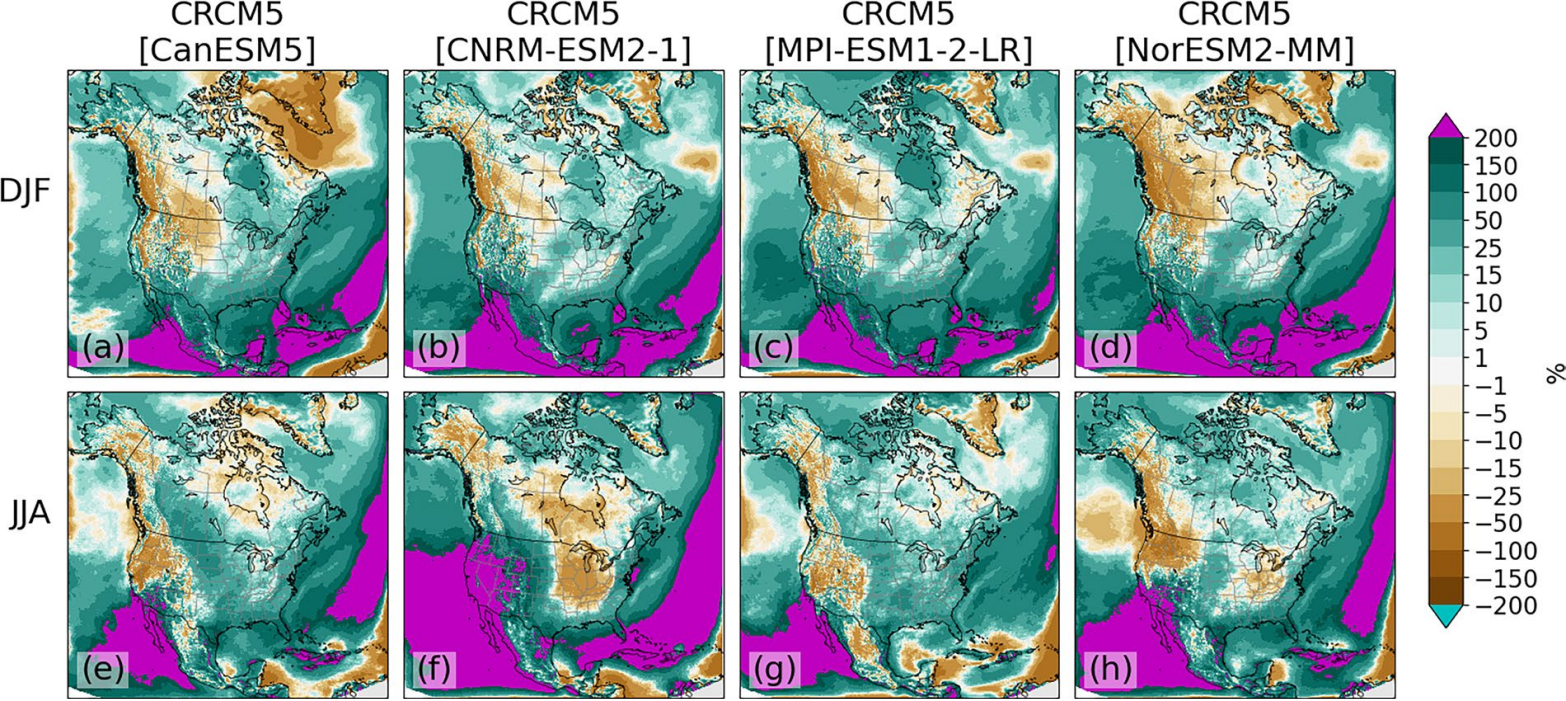
As in Fig. 3, but for relative precipitation bias (%).

Among all simulations, wet biases are present over most regions in winter except over western Canada and portions of the northwestern U.S. (Fig. 4a–d). The CRCM5[CanESM5] simulation also has a region of dry bias over Greenland and the Labrador Sea (Fig. 4a). All simulations also present strong (>200%) wet biases off the Pacific coast of Mexico and California during all seasons. All simulations except CRCM5[MPI-ESM1-2-LR] also have strong wet biases from the Caribbean northeastward across the Atlantic that are strongest during summer. Preliminary analyses (not shown) suggest these biases may be partially related to tropical cyclone development.

**Fig. 5:**
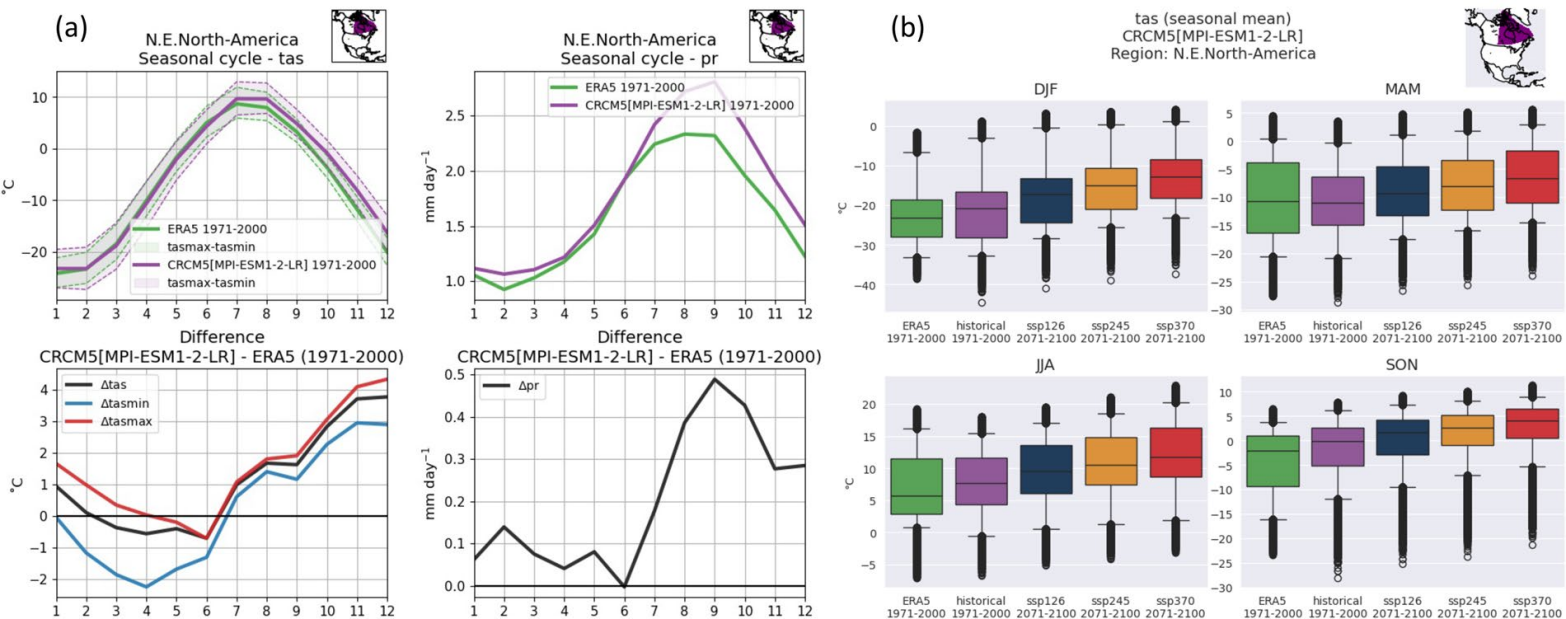
Examples of two types of figures used for evaluation of CRCM5 simulations. Seasonal cycle graphics showing the monthly average tas (solid lines) and the tasmax-tasmin range (shaded area bounded by dashed lines) (**a**, left panel) and pr (a, right panel) for the northeastern North America (NEN) region for ERA5 (green) and CRCM5 driven by MPI-ESM1-2-LR (purple) for 1971–2000. Bottom plots of each panel show the values from the CRCM5 simulation minus ERA5 values for each variable. Box plots (**b**) display distributions of tas among NEN grid points in ERA5 and the historical, SSP1-2.6, 2-4.5 and 3-7.0 CRCM5[MPI-ESM1-2-LR] simulations for the four seasons.

In addition to the examination of spatial patterns, seasonal cycles of basic variables are also explored to ensure that the month-to-month meteorological variability of different climatic regions is well represented in the model. The nine Intergovernmental Panel on Climate Change (IPCC) climate reference regions^22^ covering the model domain over land are used for this analysis. Figure 5a presents an example of the type of analysis performed for one simulation and region, CRCM5[MPI-ESM1-2-LR] for northeastern North America (NEN). In this example, the seasonal cycles of tas, tasmax and tasmin are well represented, with a slight (0–2 °C) cold bias in the spring transitioning to a stronger (1–4 °C) warm bias by early winter. The seasonal cycle of pr is also well represented, with the model reproducing the expected dry season during winter and the wettest months during summer and autumn. Precipitation biases are generally small except during the wet season where biases peak at +0.5 mm day^−1^ in September.

**Fig. 6:**
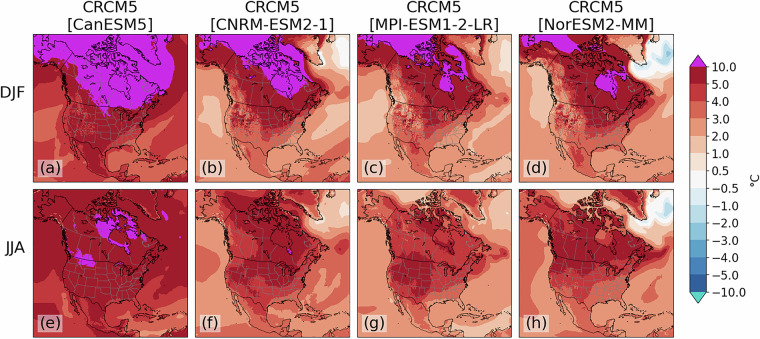
As in Fig. 3, except showing projected changes to tas from 1971–2000 to 2071–2100 in each of the SSP3-7.0 simulations.

Future climate simulations are evaluated to ensure large-scale climate change signals are physically reasonable and coherent with results from the driving GCMs themselves. Figure 6 displays the average temperature change from the late-20th (1971–2000) to late-21st century (2071–2100) for the SSP3-7.0 simulations. Projected temperature changes are correlated with the ECS of the driving GCM, with the strongest warming projected in the CRCM5[CanESM5] simulation (Fig. 6a,e). Over 10 °C of average warming is projected by this simulation across much of Canada, Greenland and Alaska in winter (Fig. 6a). Conversely, as expected because it is the driving GCM withs the smallest ECS, the weakest warming is found in the CRCM5[NorESM2-MM] simulation (Figs. 6d,h).

**Fig. 7:**
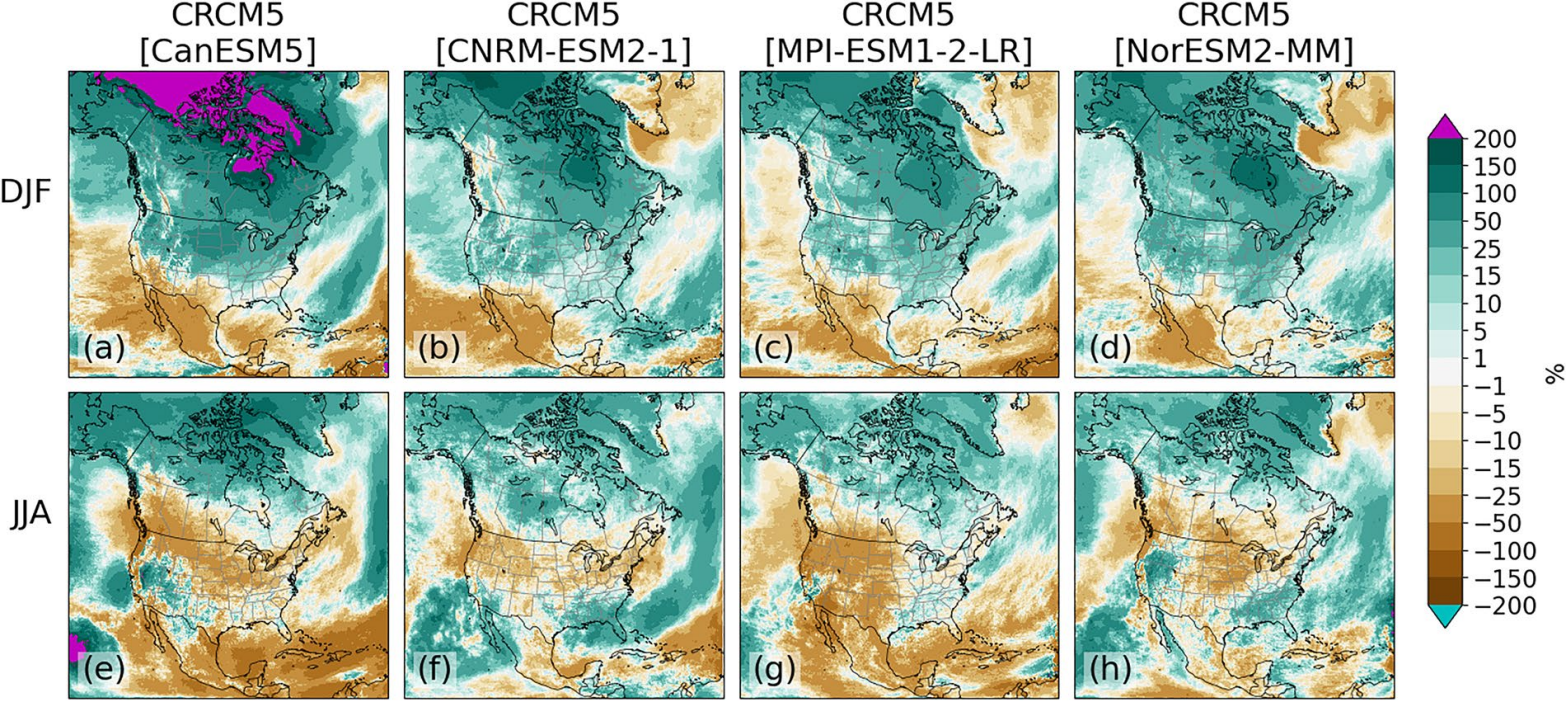
As in Fig. 6 except for relative precipitation changes.

Precipitation change signals are broadly coherent among members, particularly in winter when all members project decreasing precipitation over southwestern North America and increases elsewhere (Figs. 7a–d). Greatest increases are found over northernmost regions, with >200% increases found in the CRCM5[CanESM5] simulation over the Arctic. Summer precipitation changes are more uncertain and variable among simulations, with general agreement on decreases over the subtropics and portions of western North America (Fig. 7e–h). The CanESM5- and CNRM-ESM2-1-driven simulations also show a band of decreasing precipitation extending eastward to the Canadian Maritimes (Fig. 7e,f).

Simulations following different future scenarios are evaluated for each IPCC climate reference region to ensure that the expected behavior is found. For example, Fig. 5b presents the distribution of seasonal mean tas among grid points in the NEN region for ERA5 and the historical and three future climate simulations driven by MPI-ESM1-2-LR. As expected, the distributions shift to higher temperature with increasing climate forcing. Seasonal cycle and box plots like those in Fig. 5 for the other simulations and IPCC regions can be found in the Github repository accompanying this manuscript (see the Code availability section).

**Fig. 8:**
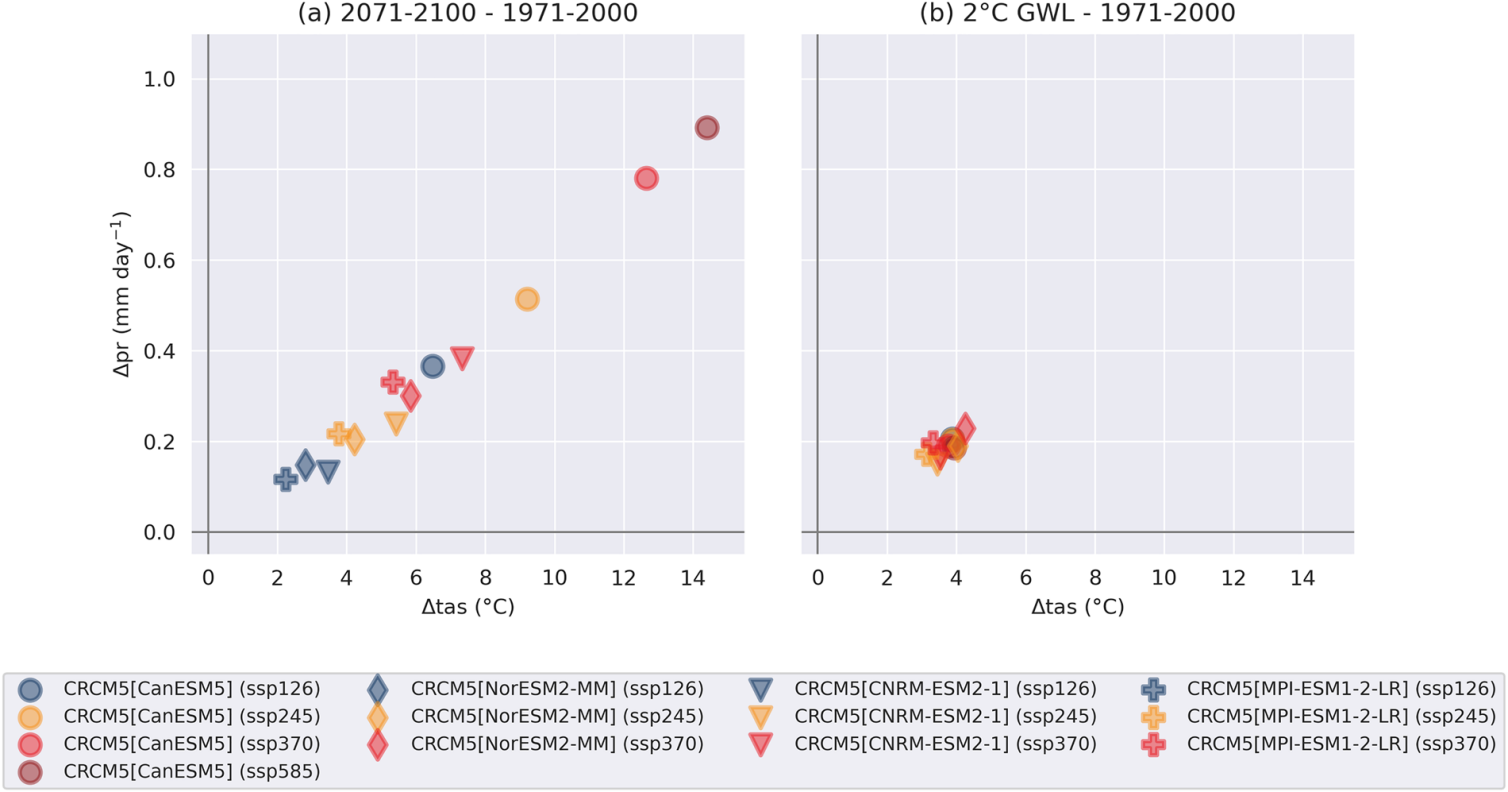
Scatter plots presenting regionally averaged projected changes in tas (°C) and pr (mm day^−1^) for NEN for 1971–2000 to 2071–2000 (**a**) and for 1971–2000 to the 30-year period during which the +2 °C global warming level (GWL) is reached **B**. **a** contains all completed simulations of the ensemble, while **b** includes the subset of nine simulations that attain this global warming level by 2100.

Finally, simulations driven by different GCMs and following different SSPs are compared through an examination of projected regional mean changes in tas and pr. For the NEN region, a strong linear relationship between these two variables is identified (Fig. 8a). As expected, simulations with the strongest forcing (SSP3-7.0 and 5–8.5) and driving GCMs with the strongest climate sensitivity (CanESM5) produce the largest changes.

In addition to evaluating changes over fixed temporal periods, we evaluate changes at the centered 30-year periods when various global warming levels (GWLs) are attained, following the technique of Nikulin *et al*.^23^. Patterns of tas and pr changes at a given GWL (not shown) are more similar than those presented previously for end-of-century changes. Over most regions, use of GWLs reduces the uncertainty between simulations related to climate sensitivity and scenario and transforms it into uncertainty related to the years of attainment of the GWL. For example, in NEN, a global warming of +2 °C relative to the 1850–1900 pre-industrial climate corresponds to a regional warming of 3.1–4.4 °C and an increase in precipitation of 0.15–0.24 mm day^−1^, relative to 1971–2000, among the nine ensemble members that reach this GWL (Fig. 8b). A table showing the time periods corresponding to different warming levels for each of the driving GCMs is available at 10.5281/zenodo.17465777^18^.

Among these nine simulations, variability for changes from 1971–2000 to 2071-2100 is much larger, ranging from 3.8–14.4 ^°^C of warming and 0.22–0.89 mm day − 1 of wetting (Fig. 8a).

Given this information, and considering that the set covers a wide range of ECS values, ranging from 2.54 K to 5.64 K, various climate trajectories are represented, allowing users to explore them independently in order to evaluate plausible future scenarios. Although the high ECS of CanESM5 has been deemed unlikely (see^24^), it remains useful as part of a narrative approach to examine potential outcomes in very warm scenarios for North America. Given these different climate trajectories, global warming levels (GWLs) should be used as a basis for comparison across simulations.

## Usage Notes

The CRCM5-CMIP6 simulation ensemble is available through a THREDDS Data Server. NetCDF files can be downloaded through the afforementioned link with http server access. An easy way to access the data is through NcMLs. NcMLs are aggregations of NetCDF files that can be accessed using the *xarray* python library (https://docs.xarray.dev/en/stable/)^25^ and the OPeNDAP protocol. We suggest using the following workflow to access the data using OPeNDAP:

- Select an NcML corresponding to the desired simulation.
- Select the OPeNDAP access.
- Use the data URL (url) in your access call: xarray.open_dataset(url, decode_timedelta=False, chunks={'time': 250, 'rlat': 50, 'rlon': 50})

For further analysis, the *xclim* (https://xclim.readthedocs.io)^26^ and *xscen* (https://xscen.readthedocs.io)^27^ packages can be used following data analysis examples described on the Power Analytics and Visualization for Climate Science (PAVICS) platform (https://pavics.ouranos.ca). Examples include data subsetting, computing climate indices and other procedures.

## Data Availability

The reference associated with the CRCM5-CMIP6 simulation ensemble is 10.5281/zenodo.17465777^18^. All data are available through a permissive CC-BY 4.0 license. The dataset are available at • for monthly • for daily • for 3-hourly • for hourly

## Code availability

The code to reproduce the figures from this paper is available in the release CRCM5-CMIP6 v1.0.7 (https://github.com/Ouranosinc/CRCM-CMIP/releases/tag/v1.0.7) of the CRCM-CMIP repository (https://github.com/Ouranosinc/CRCM-CMIP). Simulation data used to produce the figures from this paper is available through the 10.5281/zenodo.17465777 zenodo repository of the CRCM5-CMIP6 ensemble (10.5281/zenodo.17465777).
