# Correction: The Ouranos CRCM5-CMIP6 ensemble: A dynamically downscaled ensemble of CMIP6 simulations over North America

**DOI:** 10.1038/s41597-026-06772-9

**Published:** 2026-02-06

**Authors:** Dominique Paquin, Christopher D. McCray, Charles B. Gauthier, Michel Giguère, Olivier Asselin, Pascal Bourgault, Marie-Pier Labonté, Dominic Matte

**Affiliations:** Ouranos, Montréal, Québec Canada

Correction to: *Scientific Data* 10.1038/s41597-025-06289-7, published online 12 December 2025.

In the version of the article initially published, Figs. 3–5 were incorrectly ordered and have now been amended so that the original Fig. 4 is now Fig. 3, the original Fig. 5 is now Fig. 4 and the original Fig. 3 is now Fig. 5. This correction has been made to the HTML and PDF versions of the article.

